# Multivariate Gas Sensor E-Nose System with PARAFAC and Machine Learning Modeling for Quantifying and Classifying the Impact of Fishing Gears

**DOI:** 10.3390/s26010006

**Published:** 2025-12-19

**Authors:** Vinie Lee Silva-Alvarado, Jaime Lloret

**Affiliations:** Instituto de Investigación para la Gestión Integrada de Zonas Costeras, Universitat Politècnica de València, Carrer del Paranimf 1, 46730 Grao de Gandia, Valencia, Spain; jlloret@dcom.upv.es

**Keywords:** E-nose, PARAFAC, Machine Learning, fishing gear impact

## Abstract

**Highlights:**

This research addresses critical gaps in the real-time quality assurance of the hydrobiological industry by developing a novel monitoring methodology. Accordingly, our findings are structured around the following key questions:

**What are the main findings?**

- A novel methodology integrating MOSR sensors, PARAFAC, and Machine Learning was implemented for real-time quality monitoring.
- The L_2_ Norm metric successfully quantified metabolic stress deviation, establishing a data-driven distinction between various fishing gears.

**What are the implications of the main findings?**

- The methodology provides an objective tool for seafood traceability and quality differentiation.
- Generating gear-specific stress profiles can be utilized as a robust standard to support modern sustainability and food quality certification schemes.

**Abstract:**

The quality of seafood is intrinsically linked to the accumulated history of stress, feeding, handling, and physical damage imposed by the fishing gear employed. This study proposes an innovative methodology using an E-nose sensor. The study species was *Sparus aurata*. Eight fishing gears were studied. The methodology integrates Parallel Factor Analysis (PARAFAC) for impact quantification and Machine Learning (ML) for classifying the fishing gear of origin. Longline was established as the method with the lowest deviation. The impact hierarchy, from highest to lowest deviation, is as follows: Aquaculture 50.61% (95% CI: 34%, 68%), Purse seine 37.92% (95% CI: 22%, 54%), Trawl 35.92% (95% CI: 21%, 51%), Gillnet (three panels) 27.69% (95% CI: 14%, 41%), Gillnet (single panel) 24.63% (95% CI: 9%, 40%), Gillnet (two panels) 18.12% (95% CI: 4%, 31%) and Hook and line 1.36% (95% CI: −10%, 13%). For the classification task, 33 ML models were evaluated. Subspace KNN model yielded the best results with an accuracy of 97.14% in the validation and 98.08% in the testing, using 35 variables. Using 10, 15, 20, 25, and 30 variables, an accuracy higher than 85% was achieved. These results demonstrate the high precision in fish traceability by exploiting the sensor response profile left by each fishing gear.

## 1. Introduction

The fishing industry, in its diverse modalities such as artisanal, industrial, or aquaculture, plays a fundamental role in global food supply, economic development, and the culture of numerous communities. However, the type of fishing gear or aquaculture system used not only determines the sustainability of the catch but also its dynamic quality, which is intrinsically linked to the accumulated history of stress, physical damage, and handling [1,2,3]. Each method implies different degrees of manipulation [4], exposure to contaminants [5], stress levels [6], and post-capture handling times [7], factors that trigger physiological responses which manifest as a “chemical signature” of the fish, making it a silent carrier of its history [6,8].

Fishing gears such as Hook and line, Gillnet, Purse seine, Longline or Trawl influence the selectivity, degree of stress, handling, exposure to contaminants, and physical damage suffered by the fish [4,6]. In parallel, aquaculture introduces other variables that affect the fish composition. The use of antibiotics, antiparasitics, growth promoters, and industrial feeds can alter their metabolism, leaving internal chemical residues that reflect their artificial environment [9,10].

Given that both capture and rearing methods lead to chemical changes in the fish’s composition [5,11], effective monitoring tools are required to verify quality and provenance. However, the current tools available to monitor these effects are generally costly and inaccessible. Techniques such as mass spectrometry [12] or chromatography [13] require specialized laboratories, trained personnel, and prolonged analysis times—resources that are not always available in fishing communities, local markets, or small processing plants [14]. Therefore, it is necessary to develop a solution that is fast, portable, and accessible.

In order to address this need for accessible and portable analytical tools, a highly promising approach lies in electronic noses (E-noses), devices that capture the volatile profile released by fish. This profile directly mirrors metabolic stress and the early stages of spoilage, providing objective parameters for assessing fish quality [15]. However, the scientific literature has not yet clearly established to what extent different fishing gears (trawls, longlines, purse seines, etc.) influence the final quality of seafood products [16]. For this reason, it is essential to combine chemometric analysis, sensor technologies, and direct comparisons between fishing methods [17,18]. This integration would enable much stronger traceability, more reliable quality control, and truly meaningful eco-certifications [18,19,20,21]. Moreover, by applying Machine Learning (ML) algorithms [22] we can accurately classify the capture method, the freshness status of the fish, and even detect potential contaminants [23]. This represents a tangible, readily implementable opportunity to enhance quality control and ensure credible sustainable certification in a sector that currently lacks accessible, objective tools [15].

In response to these challenges, the primary objective of this study is two-fold: first, to quantify the impact of fishing gears [15,23] and classify them correctly through an objective metric, and second, to generate a highly accurate classification model capable of determining the capture method of a fish based on the sensor response profile with respect to the intrinsic basal profile. The species studied is *Sparus aurata* [24]. We achieve this by integrating an Electronic Nose (E-nose) system with Parallel Factor Analysis (PARAFAC) for data decomposition, allowing us to isolate and quantify the chemical stress signature of each fishing method. The resulting classification model demonstrates high accuracy in distinguishing between the capture methods, providing a robust, cost-effective, and scalable tool for traceability and sustainable quality control in the seafood value chain.

The rest of the study is divided into five sections. Section 2 details the most relevant reported studies, whereas Section 3 describes Materials and Methods. Results are presented in Section 4, followed by Discussion in Section 5 and Conclusions and future perspectives in Section 6.

## 2. Related Works

The quality and chemical characteristics of fish can be influenced by post-capture factors and by the conditions inherent to the fishing gear, although this last aspect has been little explored. The quality and chemical characteristics of fish are intrinsically linked to the inherent conditions of the procurement method, an aspect that still requires further exploration. Research has revealed that the stress caused during the capture process by certain fishing gears can induce a biological response that affects the chemical composition of the fish muscle, altering the pH and generating lactic acid, which impacts texture and flavor [25]. Broader reviews have explored how fishing gear influences the physiological and biochemical parameters of fish. Specifically, Sureda et al. [26] reported how different fishing methods impact the biochemical properties of the muscle and filet quality. Santos et al. [27] reported that discards of *Merluccius merluccius* constituted 42% of the total catch of Gillnet, but only 7% of longlines targeting the same species. Toledo-Guedes et al. [28] quantified a mortality rate of 27.3% for *Pollachius virens* caught with gillnet, while 100% of the fish caught with jigging were hauled alive onboard. Furthermore, Badaoui et al. [29] using metabolomics, found a difference in the physiological response that differentiates wild-caught and farmed *Sparus aurata*. This points to a real link between how the fish is caught and its quality after capture. To tease apart the influence of different factors on the chemical “fingerprint” of the fish, researchers have turned to a range of multivariate analysis and mathematical modeling tools. For example Lai J. et al. [30] used elemental fingerprinting to successfully distinguish between different populations of gilthead sea bream (*Sparus aurata*). In metabolomics, Bodin et al. [31] applied NMR-based metabolic profiling to assess the quality of tuna, relying on Partial Least Squares Discriminant Analysis (PLS-DA). They showed that these methods can sharply separate samples based on population and storage conditions, while also pinpointing exactly which compounds drive those differences. Taken together, these studies strongly suggest that the type of fishing gear leaves a detectable, quantifiable chemical trace in the fish—something we can (and should) use for traceability.

Turning the raw, messy signals from an electronic nose into something meaningful requires smart data handling. That is where Parallel Factor Analysis (PARAFAC) really shines in chemometrics. Yan et al. [18] reviewed feature-extraction techniques for E-noses and concluded that PARAFAC (along with other multivariate methods) is one of the best ways to pull clean, reliable, and non-redundant information out of sensor arrays. PARAFAC is not just for gases either: Rosa et al. applied it to near-infrared spectroscopy data to track the thermal degradation of rice oil [32] and soybean oil [33]. They were able to isolate and quantify two distinct factors—the formation of oxidation products and the protective effect of antioxidants—proving how powerful the technique is at untangling overlapping signals. Analogously, Liu et al. [34] used UV-vis and fluorescence spectroscopy with PARAFAC, successfully characterizing four Dissolved Organic Matter (DOM) ranks. PARAFAC’s ability to isolate and quantify pure causal ranks within complex matrices (such as the stress signal) is the basis that allows this study to go beyond simple classification to establish an impact metric.

Recent advances in gas sensor arrays and ML techniques have enabled more accurate assessments of such changes. Numerous studies have applied and recommend diverse types of analysis models in the food industry applied to E-noses. Reference [19] developed a gas sensor system for real-time monitoring and detection of fish quality and deterioration with high precision. Wijaya et al. [23] proposed a method for detecting seafood quality using E-noses optimizing their performance through hyperparameter tuning. In a similar context, Yavuzer [15] determined fish quality parameters with a low-cost E-nose. Silva et al. [24] used an array of Resistive Metal Oxide Semiconductor based (MOSR) sensor modules to discriminate between different fish species and estimate time without refrigeration. Other authors focused on the development of optical sensors. Balım et al. [35] developed a method for classifying fish freshness using laser reflectance and RGB image features based on deep learning to discriminate between fresh and deteriorated products. Research such as that by Shinoda et al. [36] has demonstrated the feasibility of wireless optical biosensors for monitoring physiological stress indicators like glucose in real-time.

The current literature confirms the validity of using PARAFAC for tensor decomposition and rank isolation, establishes the existence of a chemical stress footprint in fish, and supports the high capacity of MOSR sensors for the detection of volatile organic compounds (VOCs) [37]. Nevertheless, a significant gap persists. To our knowledge, there is currently no methodology that integrates MOSR sensor array technology with PARAFAC tensorial decomposition to rigorously quantify the impact of various fishing gears and classify the origin of the fishing gear. The novelty of this work lies in the proposal of a new impact metric derived from PARAFAC to determine which fishing gear presents the greatest variation with respect to the intrinsic basal profile of a fish species and, subsequently, to correctly classify the fishing gears using ML techniques. These differences reflect factors such as induced stress, contamination, or the specific interventions of aquaculture.

## 3. Materials and Methods

This section presents a detailed explanation of the sensor employed, the data analysis and processing to evaluate the impact of fishing gear on the intrinsic basal profile of *Sparus aurata*, and the parameters established for the classification of fishing gears.

### 3.1. Sensor

**Figure 1:**
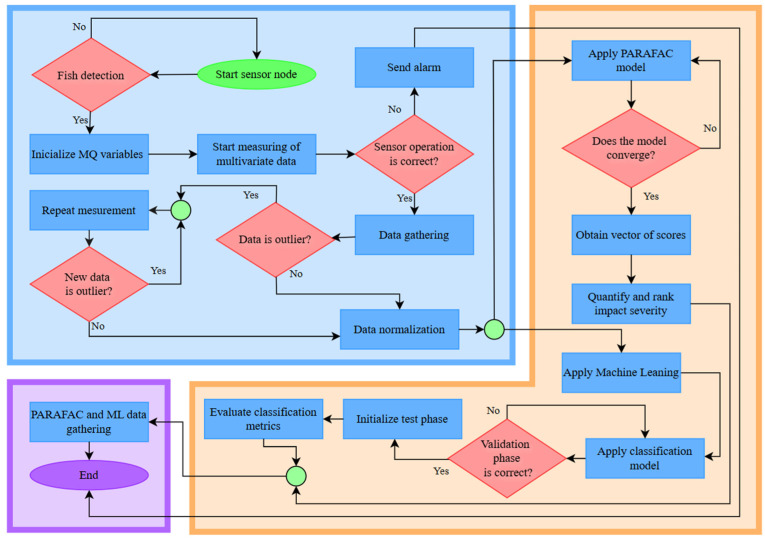
Proposed node operation. The light blue shaded area is the Data acquisition and preprocessing stage. The orange shaded area is the Chemometric and Machine Learning Analysis stage. The purple shaded area is the Communication and Transfer Data stage. Green dots indicate flow connectors linking different processes in the diagram.

A gas sensor setup based on MOSR technology, widely used for VOC detection, was employed. The 35 variables extracted from the MQ sensor modules are detailed in Table 1. The algorithm governing the processes is shown in Figure 1. 

### 3.2. Data Collection

The sampling was performed individually for each fishing gear. *Sparus aurata* samples were obtained directly from fishermen upon their arrival at the port. The fishing areas are located in the Western Mediterranean Sea. The port of landing was Gandía. The time elapsed from sample capture until landing was considered an intrinsic part of the accumulated impact of the fishing gear. This approach was based on the fact that each distinct gear type inherently dictates its own operational protocols, handling complexities, and retrieval times, making this duration an inseparable, characteristic variable of the classification factor. On the other hand, the subsequent time, from port reception until the moment of analysis, was standardized and uniform for all samples, thus eliminating this variable as a differentiating or confounding factor. To mitigate sensor drift and environmental effects, the sensor response was normalized and recorded as the dynamic ratio response ($\left|{R_{0}-R}_{S}\right|/R_{0}$), where $R_{0}$ is the stable baseline resistance recorded in a controlled clean air environment [37]. Additionally, new MQ sensor modules were employed for each sampling session to eliminate long-term drift accumulation.

**Figure 2:**
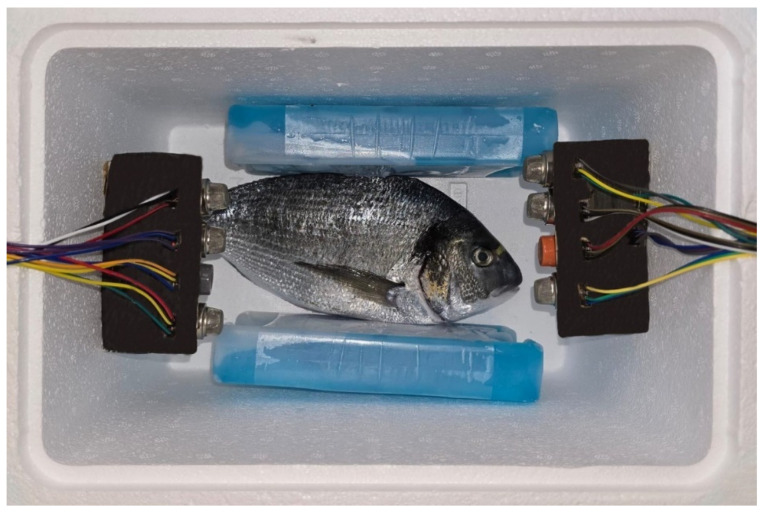
Top view of the measurement chamber with the MQ sensor modules.

The eviscerated sample was placed (as seen in Figure 2) for 10 min before measurement to condition the chamber. The sample was eviscerated to eliminate the variability of the microbial load. The chamber was hermetically sealed with film. The temperature inside the chamber was maintained at 10 °C throughout all sampling. The sensor was configured to record readings at a frequency of 0.65 min, covering a total period of 1.5 days (36 h). This extended, high-frequency measurements was specifically designed to capture the kinetics of release and the detailed evolutionary profile of post-capture VOCs. For each fishing gear category, 10 biological replicates were collected. Sample weights were 279±20 g with a 95% CI; this controlled biometric range was implemented to minimize the physiological variability related to mass, thereby supporting the assumption that weight variations had no significant impact on the sensor responses.

### 3.3. Preprocessing

Each dataset corresponded to a single fishing gear. It consisted of the measurement of 35 sensor variables along 2600 time instances. For the analysis, these data were structured into a three-dimensional tensor described in Equation (1).

(1) $$X\in R^{I\times J\times K}$$

where *I* represents the time instances, *J* represents the sensor variables, and *K* represents the fishing gears. Preprocessing was carried out using Python 3.9 with the *pandas* library. A cleaning and standardization process was performed for each data file, consisting of:

- Reading: CSV files were imported, and non-numeric values were converted to NaN.
- Missing Value Imputation: Missing values were imputed using a temporal window algorithm. For each missing value, up to gamma previous valid observations and up to omega subsequent valid observations are considered. If both windows exist, the mean of each window ($\mu_{prev},\;\mu_{next}$) and the relative difference (δ) are calculated as explained by Equation (2).

(2) $$\delta =\frac{\left|\mu_{prev}-\mu_{next}\right|}{max\left(\left|\mu_{prev}\right|,\;\left|\mu_{next}\right|,\varepsilon \right)}$$

If δ≤τ (tolerancia relativa) (relative tolerance), the imputed value is obtained as the weighted mean of the two windows; if δ>τ linear interpolation is applied between the last previous observation and the first subsequent one. If neighbors from only one side exist, the mean of the available window is used; if the series completely lacks valid observations, the column median is used as a fallback value. In this work, we assume γ=10, τ=0.5 and $\varepsilon =10^{-12}$ which is a numerical tolerance constant used to prevent division-by-zero errors. Additionally, a binary mask (0/1) was generated to identify the imputed positions, which

Outlier detection and treatment: Extreme values were identified using the interquartile range (IQR). The interval was defined according to Equation (3).

(3) $$I=\left[Q_{1}-\alpha \cdot IQR,Q_{3}+\alpha \cdot IQR\right]$$

where α=1.5 is a sensitivity factor. Outliers were marked as atypical, and a missing value imputation process, as previously described, was performed; this filtering step is crucial to avoid overfitting to sensor noise and transient artifacts.

The data from each sensor were standardized (StandardScale) so that each series had a mean of 0 and a standard deviation of 1.

### 3.4. Data Processing

The software employed were Python 3.9 and MATLAB R2025a. 35 variables obtained from the MQ sensors were processed. To determine the impact of the fishing gear, 50 different ranks (r) of PARAFAC were studied with 100 distinct initializations, creating a total of 5000 models. 33 ML models were trained to classify the fishing gears, with 65% of the readings assigned for training, 25% for validation, and the remaining 10% for testing the models. The models obtained with the training dataset were evaluated using both the validation dataset and the testing dataset.

#### 3.4.1. Tensorial Analysis (PARAFAC) for Impact Assessment

PARAFAC was employed to decompose the data tensor X into a set of latent ranks representing the underlying patterns of the chemical signature. PARAFAC allows the tensor to be modeled as the sum of vector products and enables the isolation of contributions from each dimension independently. The model is expressed mathematically in Equation (4).

(4) $$X_{ijk}\approx \sum_{r=1}^{R}\omega_{r}.\alpha_{ir}.b_{jr}.c_{kr}+E_{ijk}$$

where $X_{ijk}$ is the element of the tensor at position (*i*, *j*, *k*). $\omega_{r}$ is the weight of the r-rank. $\alpha_{ir}$, $b_{jr}$ and $c_{kr}$ are the elements of the normalized factor matrices $A\;\left(time\right)\in R^{I\times R}$, $B\;\left(sensors\right)\in R^{J\times R}$ and $C\;\left(fishing\;gear\right)\in R^{K\times R}$, respectively. $E_{ijk}$ is the residual or error unexplained by the model.

The model was executed using the tensorly 0.8.1 library. The number of ranks r was chosen from 1 to 10. 100 distinct seeds were employed for initialization. These values were considered to study the general trend of the models at different numbers of ranks and different initializations. normalize_factors = True was set in the PARAFAC function; the magnitude of each rank was stored in the vector $\omega_{r}$, esto permite interpretar la importancia de cada factor

#### 3.4.2. Determination of the Number of Ranks (r)

The Cumulative Explained Variance (${\mathrm{VE}}_{C}$) was used as the main criterion and is expressed in Equation (5). A range of r=1 to r=50 was evaluated across 100 random initializations (seeds) for each r. This procedure was performed to identify the elbow in the explained variance curve. The inflection point will be selected as the r value that maximizes the explained variance while minimizing the inclusion of residual noise.

(5) $$VE_{C}=100\times \left(1-\frac{{\left\|X-\hat{X}\right\|}_{F}^{2}}{{\left\|X\right\|}_{F}^{2}}\right)$$

where X represents the original data tensor $X\in R^{I\times J\times K}$. $\hat{X}$ is the reconstructed tensor estimated by the PARAFAC model with r ranks. ${\left\|\cdot \right\|}_{F}^{2}$ is the squared Frobenius norm.

#### 3.4.3. Quantification of the Fishing Gear Impact

To quantify the impact of each fishing gear, the magnitude of the deviation of its response profile for each sensor variable was evaluated with respect to the intrinsic basal profile of *Sparus aurata*. This deviation was quantified using the Euclidean norm ($L_{2}$ norm) of the contributions of the rank (r) that determines the optimal dimensionality.

The impact metric for each fishing gear (k) is defined as the total magnitude of its accumulated contribution across all selected optimal latent ranks (r). To this end, the contribution of rank (r) to a specific fishing gear (k) is modeled as the product between the rank weight ($\omega_{r}$) and the fishing gear factor ($c_{kr}$). Thus, the point impact ($I_{k}^{\left(s\right)}$) of a fishing gear k for a seed index (s) is calculated as the $L_{2}$ norm of the contribution of the r ranks, according to Equation (6).

(6) $$I_{k}^{\left(s\right)}=\sqrt{\sum_{r=1}^{R}{\left(\omega_{r}.c_{kr}\right)}^{2}}$$

Subsequently, to reduce the variability associated with different initial conditions, the impact of each fishing gear was averaged over 100 distinct seeds for each r value.

#### 3.4.4. Classification of Fishing Gears

The models used to classify the fishing gears are detailed in Table 2. Each method offers different benefits, especially in terms of adapting to distinct patterns and complexity levels in the data. The metric employed to measure classification performance is accuracy, given its frequent use by numerous authors.

## 4. Results

This section presents the results. First, a summary of the general results from all tests is provided. Subsequently, the performance of the developed models is discussed.

### 4.1. Fishing Gear Impact

With PARAFAC, 5000 models were studied, comprising 50 different rank (r) values, each with 100 distinct initializations, to estimate the appropriate number of ranks for the impact calculation.

**Figure 3:**
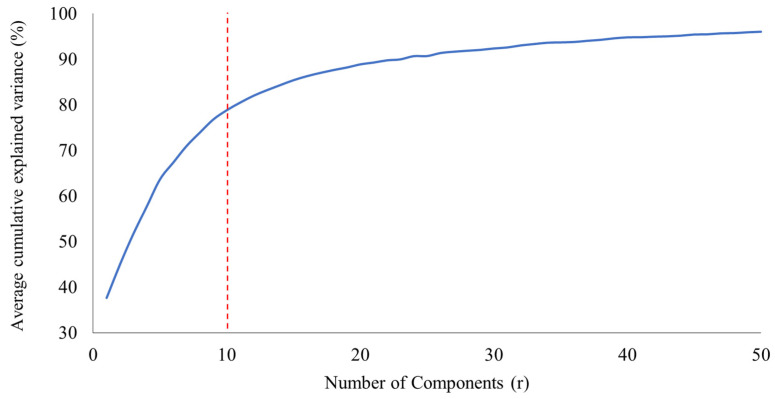
Average cumulative explained variance (%) as a function of the number of ranks (*r*).

The calculation covered r=1 up to r=50 ranks, across 100 distinct seeds (initializations). The results of the average Cumulative Explained Variance are presented in Figure 3, illustrating the progressive variance gain upon incorporating each rank.

The analysis revealed that the model with r=1 explains approximately 37.6% of the variance. The variance increases significantly, reaching 78.79% with r=10 ranks. Although the explained variance climbs to a maximum of 96.0% with r=50 ranks, the small percentage gain after r=10 suggests that the model begins to capture low-level variability and noise. Consequently, the number of ranks at the inflection point was selected for the calculation of the impact metric, optimizing the balance between explained variance and model stability.

**Figure 4:**
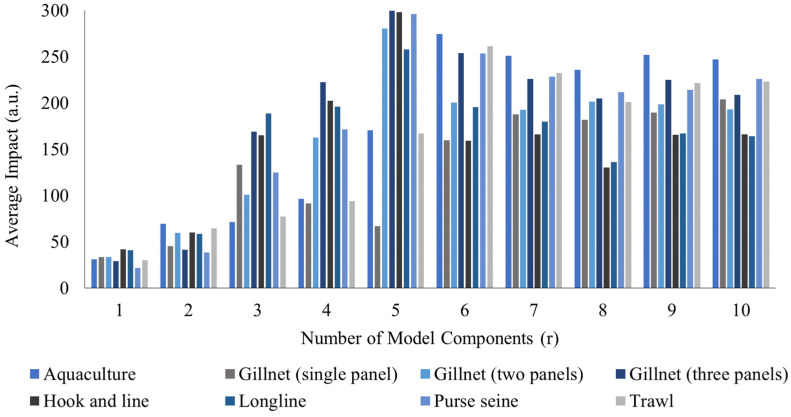
Stability of the Impact Metric as a Function of Model Complexity (r).

For each number of ranks (r) up to 10, the average impact over the 100 initializations (seeds) was calculated. The result of this first stage is the impact metric used to plot the stability in Figure 4. The initial results for r=1 and r=2 are low and unstable, as the model still fails to adequately separate the basal component from the variation components. Starting from r=6 up to 10, the trend of the impact values for each fishing gear stabilizes. This inflection point indicates that this number of ranks is sufficient to isolate the main patterns (basal profile, feeding, stress, handling, damage, and metabolic alteration). The hierarchical order of impact from this point does not vary significantly, which would explain why they do not depend on the exact choice of r in the upper range.

### 4.2. Quantification of the Global Average Fishing Gear Impact

To obtain the definitive impact metric r = 10 was chosen because it corresponds to the ‘elbow’ inflection point of the explained variance, capturing 78.79% of the total system variation. The global average impact (${\bar{I}}_{k}$) over the 100 initializations was calculated.

**Figure 5:**
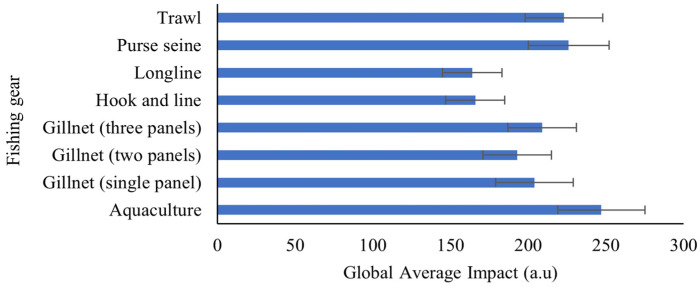
Global Average Impact (${\bar{I}}_{k}$) for Fishing Gear. Arbitrary units (a.u).

Figure 5 presents the global average impact (average $L_{2}$ norm) of the eight investigated fishing gears with a 95% confidence interval. Samples obtained from aquaculture showed the highest average impact, with 247±28 a.u. This reflects a significant deviation in the intrinsic basal profile of *Sparus aurata*, which is attributed to the artificial diet and confined environment rather than acute capture stress. Purse seine and trawl fishing occupy the second and third places for highest impact, respectively, consistent with the high physical stress associated with crowding and abrasion [1] showing a mean $L_{2}$ norm of 226±26 a.u. and 223±25 a.u., respectively. Gillnet (three panels), Gillnet (single panel) and Gillnet (two panels) reached a mean $L_{2}$ norm of 209±22 a.u. 204±25 a.u. and 193±22 a.u., respectively. Longline fishing was the gear with the lowest recorded impact, followed by Hook and line, with a mean $L_{2}$ norm of 164±19 a.u. and 166±19 a.u., respectively. This may be due to these methods being individual capture techniques that minimize striking and stress due to crowding.

Longline, with an $L_{2}$ norm of 164±19 a.u., was established as the basal profile of minimum impact. It is assumed that it is not possible to find a sample with a lower impact than those obtained by this fishing gear for *Sparus aurata*. Based on this value, the relative percentage difference was calculated for each gear. A severity scale is established and presented in Table 3 which validates that the impact severity is grouped into physiologically coherent categories [3,26,38].

### 4.3. Classification Learning of Fishing Gear

Features derived from the MQ sensor responses were used for the classification of the fishing gears. The accuracy of the models was evaluated across subsets of 10, 15, 20, 25, 30, and 35 variables.

**Figure 6:**
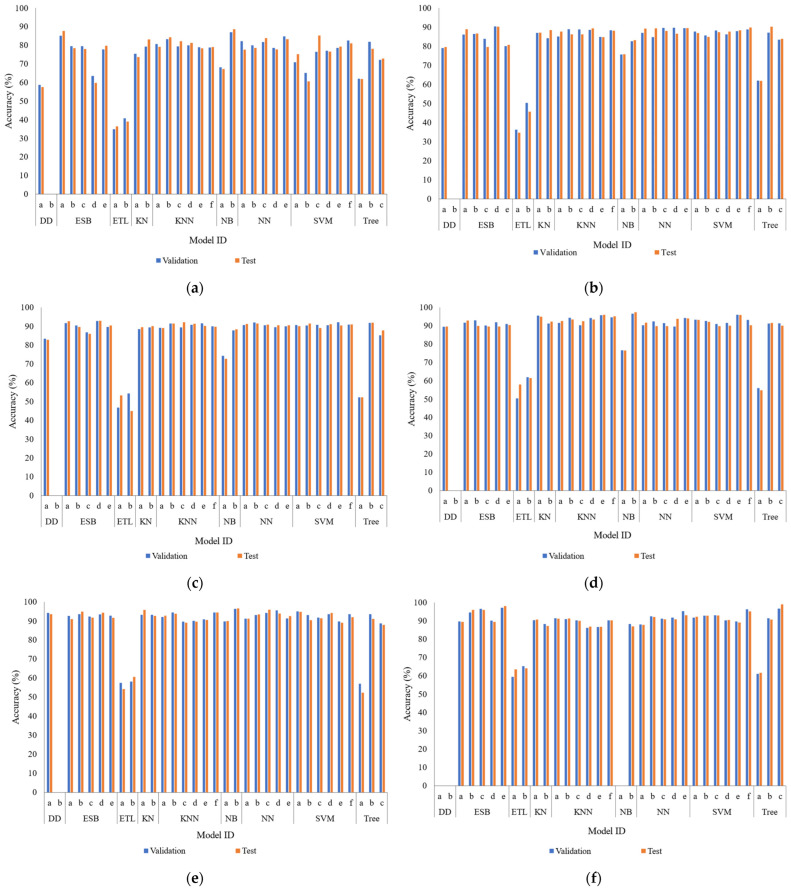
Comparative Performance of Machine Learning Models in Fishing Gear Classification. (**a**) 10 variables (MQ-4 and 7). (**b**) 15 variables (MQ-4, 7 and 8). (**c**) 20 variables (MQ-4, 6, 7 and 8). (**d**) 25 variables (MQ-2, 4, 6, 7 and 8). (**e**) 30 variables (MQ-2, 4, 5, 6, 7 and 8). (**f**) 35 variables (MQ-2, 3, 4, 5, 6, 7 and 8).

Using 10 variables (MQ-4 and MQ-7 sensors), the Kernel Naive Bayes model achieved an accuracy of 86.97% in the validation phase and 88.65% in the test phase (see Figure 6a). Increasing to 15 variables (MQ-4, 7, and 8), the Subspace Discriminant model reached an accuracy of 90.35% in validation and 90.29% in test (see Figure 6b). With 20 variables (MQ-4, 6, 7, and 8), the Subspace Discriminant model remained first, obtaining 92.79% in validation and 92.93% in test (see Figure 6c). With 25 variables (MQ-2, 4, 6, 7, and 8), the Kernel Naive Bayes model reached an accuracy of up to 96.60% in validation and 97.36% in test (see Figure 6d). Subsequently, the set of 30 variables (MQ-2, 4, 5, 6, 7, and 8) with the Kernel Naive Bayes model showed a slight saturation, with accuracies of 96.36% in validation and 96.59% in test (see Figure 6e). Finally, by employing the entirety of the 35 MQ sensor variables (from 2 to 8), the Subspace KNN model achieved the maximum discrimination capacity, with 97.14% in the validation phase and 98.08% in the test phase (see Figure 6f). These results confirm that the use of the complete sensor array allows for the most precise identification of the fishing gears. In Figure 6a–e, the achieved accuracy is presented using various sensor arrays. The validation phase is presented in blue and the test phase in orange. The models are grouped by Model ID and organized according to Table 2.

#### Subspace KNN Classification Model

**Figure 7:**
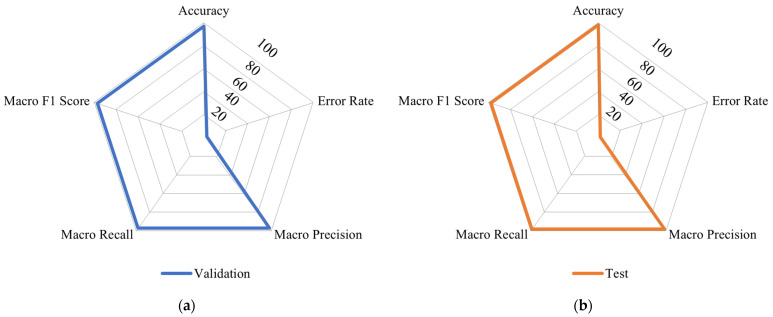
Subspace KNN Classification Model Performance: Macro Metrics in Percentage Scale Visualization using Spider Plots for (**a**) Validation and (**b**) Test Datasets.

When all 35 variables are used, the Subspace KNN model achieved the best performance with an accuracy of 97.14% in the validation phase and 98.08% in the test phase. The comprehensive performance profile, including operational efficiency and detailed metrics, is presented in Figure 7. The model demonstrated high operational efficiency: Prediction Speed 174.6 obs/sec, Training Time 205.3 s, Coder Model Size 83.61 MB.

**Figure 8:**
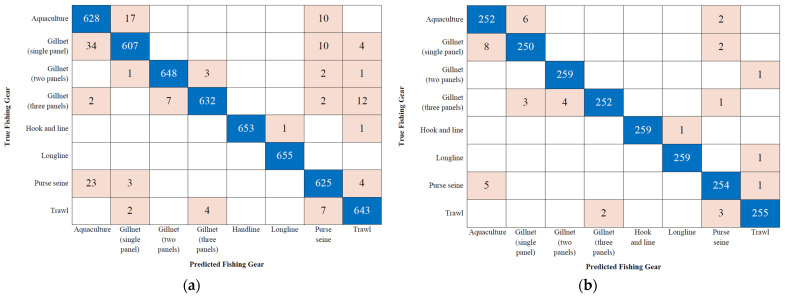
Confusion Matrices of the Subspace KNN Model with 35 Variables. (**a**) Validation Set. (**b**) Testing Set.

The errors in the confusion matrices in Figure 8 are concentrated in systematic patterns that reflect the similarity in response profiles induced by the fishing operations. The most significant confusion occurs between aquaculture and high-volume net methods (gillnet and purse seine). This error is due to the fact that both the chronic stress from confinement in aquaculture and the crowding, striking, and physical handling in nets generate comparable Volatile Organic Compound (VOC) signatures. Other errors correlate with the degree of physical damage, such as the confusion between Gillnet (three panels) and trawl, methods that induce a similar acute stress profile. In contrast, the most selective and lowest-impact methods, such as hook and line and longline, exhibit the lowest error rates, as observed in Figure 8a,b, confirming that less aggressive handling produces more distinctive and separable chemical signatures. In summary, the model successfully classifies by isolating fundamental chemical differences. Crucially, the residual errors validate that the similarity in the VOC profile is a direct consequence of the operation and the physical impact of the fishing gear, confirming the physiological clustering previously quantified by the L_2_ Norm impact metric.

**Figure 9:**
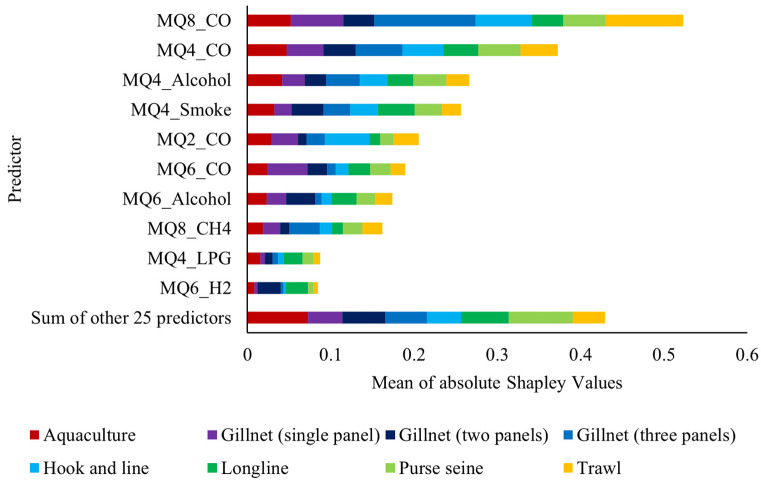
Shapley importance for Subspace KNN model with 35 variables.

Figure 9 presents the average influence of the predictive variables in the Subspace KNN model, quantified by the mean of the absolute Shapley values. This Explainable Artificial Intelligence (XAI) metric indicates the average impact of each sensor variable on the magnitude of the prediction [39] to the fishing gear classification. The high stability of this predictive hierarchy was consistently verified, as the relative ranking of feature importance remained invariant across all cross-validation folds.

The Shapley analysis reveals a clear chemical hierarchy in the model’s decision-making, which validates that the classification is based on evidence of physiological impact and post-capture handling. The most influential predictors are consistently the readings for Carbon Monoxide (MQ8_CO, MQ4_CO) and Alcohols (MQ4_Alcohol, MQ6_Alcohol). This dependence is crucial: different fishing gears impose distinct levels of physical damage and stress, which affects enzymatic release and initial pH [3]. The stress and struggle induced by certain methods (such as confinement in aquaculture or trawl) force the muscle to perform anaerobic metabolism, leading to rapid chemical changes and the release of specific Volatile Organic Compounds (VOCs). Alcohols are direct markers of this anaerobic fermentation and incipient spoilage, while CO and other gases are byproducts of metabolic stress and accelerated cellular breakdown. The model exploits this combined signature of stress/damage and the initial spoilage rate to create a robust discriminatory profile, confirming that the classification of origin is, in essence, an impact classification.

## 5. Discussion

Various studies conclude that there is a significant difference in stress levels for fish captured by distinct fishing gears [3,16,28,38,40]. However, these investigations are based on chemical analysis, mortality rates, percentage of damaged individuals, among others. The L_2_ Norm applied to the PARAFAC scores allowed us to group the impact severity into physiologically coherent categories. The fishing gear with the highest impact is aquaculture with 50.61% (95% CI: 34%, 68%). The significant deviation in aquaculture is primarily due to a chronic metabolic alteration inherent to the production system, influenced by the artificial diet and potential medication, which fundamentally modifies the composition of basal VOCs in the tissue [29]. Purse seine and Trawl reached 37.92% (95% CI: 22%, 54%) and 35.92% (95% CI: 21%, 51%), respectively, indicating a moderate to high impact. The deviation in purse seine and trawl is due to acute and severe stress from crushing and confinement [1,4,26,40], which forces anaerobic metabolism and rapid enzymatic release. This results in the accelerated production of alcohols and carbon monoxide (CO), creating VOC signatures of physical trauma and metabolic alteration similar in magnitude. Finally, the moderate impact methods are Gillnet (three panels), Gillnet (single panel), and Gillnet (two panels) with 27.69% (95% CI: 14%, 41%), 24.63% (95% CI: 9%, 40%), and 18.12% (95% CI: 4%, 31%), respectively. Their deviation correlates with stress from hypoxia and immobilization [16,28], which generates a more attenuated and different VOC signature compared to mechanical trauma. Finally, Hook and line with 1.36% (95% CI: −10%, 13%) and Longline with 0% (95% CI: −12%, 12%) show minimal deviations [27]. This is consistent with faster handling and insignificant tissue damage, which minimizes anaerobic metabolism and post-capture VOC release. Furthermore, both fishing gears are not statistically distinguishable.

In the field of classification, there are various studies utilizing E-nose on marine products. Wijaya et al. [23] achieved 99% accuracy in freshness and an R^2^ of 0.995 to predict microbiological development. Silva et al. [24] achieved 99% accuracy to classify species and an R^2^ of 0.99 to estimate the time without refrigeration. Other approaches, such as that by Balim et al. [35] achieved an accuracy of 88.44% to classify freshness. However, these investigations focus on classifying freshness or distinct species. In contrast, works focused on differentiating populations within the same species were reviewed. For example, Lai et al. with two Principal Components (PC), explained 50.2% of the total variance in the distinction of different *Sparus aurata* populations. Bodin et al. [31] managed to classify geographical origin with a predictive power Q^2^ of 0.83 and storage conditions with a Q^2^ of 0.82. The described models achieve an accuracy inferior to the proposed classification model considering the same fish species. With the proposed Subspace KNN classification model, an accuracy of 97.14% was achieved in the validation phase and 98.08% in the test phase.

The performance of the proposed system validates the efficacy of the sensors and the analysis methodology. Furthermore, the coherence of the erroneous classifications between aquaculture and high-volume fishing gears confirms that the system is measuring functional similarity in physiological stress profiles and not random errors. The system is cost-effective and does not require specialized personnel, offering a practical and affordable solution for the value chain.

## 6. Conclusions

The objective quantification of fishing gear impact is a methodological challenge that requires techniques capable of isolating subtle variation signals. Our approach based on PARAFAC decomposition and Machine Learning (ML) classification of the Volatile Organic Compound (VOC) profile, offers a robust and verifiable solution.

The results demonstrate that the procurement method that deviates the most from the intrinsic basal profile of *Sparus aurata* is aquaculture, with 50.61% (IC 95%: 34%, 68%). Those with the lowest impact were Longline with 0% (IC 95%: −12%, 12%) and Hook and line with 1.36% (IC 95%: −10%, 13%). For fishing gear classification, the Subspace KNN model achieved an accuracy of 97.14% for validation and 98.08% for testing. Using the mean of the absolute Shapley values the most influential sensors were determined to be the MQ4 and MQ8.

Despite the robustness of the proposed methodological approach, involving the PARAFAC and ML models, it is imperative to acknowledge that further research is still required to ensure the precision of the obtained results.

Future work will focus on two key areas: the scalability and validation of the methodology. Additionally, it will systematically address the influence of critical intrinsic variables, such as capture-to-port time and sample weight. First, to confirm generalizability, dedicated external validation studies are required. This involves expanding the database to include samples collected across diverse geographical basins, multiple ports and various fishing seasons to assure the model’s robustness under varying real-world conditions, as well as incorporating more species of commercial interest. It is also crucial to expand the database to include more species of commercial interest and to evaluate how water quality [21] influences the results. Second, we plan to systematically address and quantify the influence of key variables currently treated as intrinsic factors, specifically: the capture-to-port time (to decouple the fishing gear impact from temporal autolysis/spoilage effects) and weight variations, as these are critical variables for practical application. To improve robustness, drift compensation techniques and advanced preprocessing [41] will be applied to isolate the sensor response from the environmental effect. In parallel, the fusion of multimodal sensors will be explored to maintain accuracy in dynamic and volatile production environments (visible/NIR optics [42]).

## Figures and Tables

**Table 1:** MQ sensor modules utilized and their sensitivities.

Sensor	Variables
MQ-2	Hydrogen, LPG, CO, alcohol, propane
MQ-3	LPG, CO, alcohol, benzene, hexane
MQ-4	LPG, CH4, CO, alcohol, smoke
MQ-5, 6, 7, 8	Hydrogen, LPG, CH4, CO, alcohol

**Table 2:** Comparison of Classification Models.

Model ID	Preset
DD a	Linear Discriminant
DD b	Quadratic Discriminant
ESB a	Bagged Trees
ESB b	Boosted Trees
ESB c	RUSBoosted Trees
ESB d	Subspace Discriminant
ESB e	Subspace KNN
ETL a	Efficient Linear SVM
ETL b	Efficient Logistic Regression
KN a	Logistic Regression Kernel
KN b	SVM Kernel
KNN a	Coarse KNN
KNN b	Cosine KNN
KNN c	Cubic KNN
KNN d	Fine KNN
KNN e	Medium KNN
KNN f	Weighted KNN
NB a	Gaussian Naive Bayes
NB b	Kernel Naive Bayes
NN a	Bilayered Neural Network
NN b	Medium Neural Network
NN c	Narrow Neural Network
NN d	Trilayered Neural Network
NN e	Wide Neural Network
SVM a	Coarse Gaussian SVM
SVM b	Cubic SVM
SVM c	Fine Gaussian SVM
SVM d	Linear SVM
SVM e	Medium Gaussian SVM
SVM f	Quadratic SVM
Tree a	Coarse Tree
Tree b	Fine Tree
Tree c	Medium Tree

**Table 3:** Relative impact increase (95% CI range) by fishing gear.

Fishing Gear	Relative Impact Difference (95% CI Limits)
Aquaculture *	50.61% (34%, 68%)
Purse seine	37.92% (22%, 54%)
Trawl	35.92% (21%, 51%)
Gillnet (three panels)	27.69% (14%, 41%)
Gillnet (single panel)	24.63% (9%, 40%)
Gillnet (two panels)	18.12% (4%, 31%)
Hook and line	1.36% (−10%, 13%)
Longline	0.00% (−12%, 12%)

* The high percentage difference for Aquaculture reflects the cumulative effects of the rearing environment (diet, medication, density) that generate a basal metabolic profile different from that of wild samples.

## Data Availability

The data provided can be found within the article. The original contributions made in this study are included in the document; any additional inquiries can be directed to the author or authors responsible.

## Abbreviations

The following abbreviations are used in this manuscript:

PARAFAC	Parallel factor analysis
ML	Machine learning
CI	Confidence interval
